# Model Teachers or Model Students? A Comparison of Video Modelling Interventions for Improving Reading Fluency and Comprehension in Children with Autism

**DOI:** 10.1007/s10803-021-05217-z

**Published:** 2021-07-30

**Authors:** Rachael Egarr, Catherine Storey

**Affiliations:** School of Social Sciences, Education and Social Work, Queens University Belfast, 69-71 University St, Belfast, BT7 1HL Northern Ireland

**Keywords:** Fluency, Reading, Video modelling, Feedforward video self-modelling, Video self-modelling, Talking mats

## Abstract

Video modelling (VM) interventions have been used to improve the fluency of individuals with learning disabilities and reading difficulties; this study aimed to replicate these findings with autism spectrum disorder (ASD) students. Four children with ASD (aged between 8 and 15) experienced two VM interventions, across 10 sessions, during an alternating treatments design: VM using a teacher model, and feedforward video self-modelling (FFVSM) where the student acted as the model. For two participants, FFVSM was found to be an effective intervention but overall, results for both interventions were inconsistent with previous research. Talking Mats Interviews were used to include these individuals within the social validation process of behavioural research.

## Introduction

Debates about the correct definition of reading fluency, and how this relates to comprehension, have been ongoing in the field of reading research since LaBerge and Samuels (1974) model of reading development. This two-stage model suggested that reading development occurred firstly in the accuracy stage (whereby close attention is necessary for successful performance) followed by the automaticity stage (in which attention to visual and phonological aspects of texts are no longer necessary for success). They raise the concept of fluency in the finer detail of this model through the suggestion that the reader’s working memory progresses through the following process when presented with written words: Visual processing, phonological processing, semantic processing and storage in episodic memory. A fluent reader will no longer have to attend to the manual decoding of visual to semantic systems as in the accuracy stage, rather, this becomes automatic. LaBerge & Samuels assert that a reader in the automaticity stage does not have to focus intently on the subskills involved in reading (letter recognition, letter sound associations, phoneme identification, blending, etc.) but can instead remain focused on deriving meaning at the episodic level and comprehending what has been read (Biancarosa & Shanley, 2016; Cummings & Petscher, 2016).

Despite LaBerge and Samuels (1974) not specifically defining Oral Reading Fluency (ORF) it became a widely used indicator of automaticity in reading, and thus research in fluency as a key component of reading development became a targeted subject of study (Allington, 1983; Samuels, 1979). Over time, the role of prosody in reading fluency became another area of debate. While prosody was not considered a component in early theoretical models of reading fluency (LaBerge & Samuels, 1974; Nathan & Stanovich, 1991; Perfetti, 1985), educators hold ‘reading with expression’ to high esteem as a necessary and defining feature of fluent reading (Allington, 1983; Dowhower, 1991; Kuhn & Stahl, 2000; National Reading Panel, 2000; Rasinkski & Hoffman, 2003). Now recognised as the third component to fluency (along with accuracy and rate) research around prosody may contribute toward an explanation of the missing linkage between fluency and comprehension, highlighted in LaBerge & Samuel’s original theory of automaticity, which suggested that some readers may read aloud and well but give little attention to the semantics and episodic processing of text. In the case of these readers, the absence of prosody would reduce their ability to make oral reading sound like spoken language (Stahl & Kuhn, 2002) therefore rendering it unlikely that they derive meaning upon which to act from the text.

Fluency is typically measured by combining rate and accuracy to calculate words read correctly per minute (WCPM) and this figure can be used to compare participant fluency to norms such as those developed by Hasbrouck and Tindal (2006). Torgesen (2000) argues that rate and accuracy are the only components of fluency that can be reliably measured, as they provide quantitative results that can be objectively analysed. Rate and accuracy are commonly measured through timed readings (Hudson et al., 2005), where the number of words read correctly, and errors made are recorded for a set time period. Standardised fluency assessments include The Dynamic Indicators of Basic Early Literacy Skills, 6th Edition (Good & Kaminski, 2002), and The Gray Oral Reading Test, Fifth Edition (GORT-5, Wiederholt & Bryant, 2012). Measurement systems used to assess prosody to date typically rely on subjective judgements of reading, such as the National Assessment of Educational Progress Fluency Scale which requires teachers to rate student prosody on a four-point scale based on a descriptive guide (Daane et al., 2005).

While ORF is now generally accepted as reading with “speed, accuracy and proper expression” (National Institute of Child Health and Human Development, 2000), comprehension is considered a much broader construct. The simple view of reading (SVR) defines comprehension as a process which can be classified into two parts, word reading (WR) and linguistic comprehension (Hoover & Gough, 1990). However, within the SVR, linguistic comprehension is not operationally defined in terms of component skills, processes and directional relations between subskills (Kim, 2017). The Direct and Indirect Effects Model of Reading (DIER) corroborates the view that WR and linguistic comprehension are the key components in reading comprehension but specifies that these are upper-level skills that directly impact reading comprehension skills. Multiple regression analysis demonstrated that these two components would not mediate reading comprehension without the lower-level skills of working memory, vocabulary, grammatical knowledge, perspective taking and comprehension monitoring (Kim, 2017; Perfetti & Stafura, 2014). In Kim’s, 2020 expansion of the DIER model, they demonstrate that component (or lower level) reading skills can be categorised as either proximal, or distal skills. Proximal skills have direct relations to reading comprehension, whereas distal skills support proximal skills and have indirect relations to reading comprehension (Kim, 2020). Text reading fluency was found to be a proximal skill in addition to WR and listening comprehension and mediates their relations to reading comprehension due to its incorporation of WR and semantic processes (Jenkins et al, 2003; Kim, 2015).

While it is understood that reading comprehension requires engagement and interaction with written language in order to simultaneously extract and construct meaning, there is a continuing debate in the literature as to how to accurately measure comprehension, and standardised assessments have been criticised for their lack of ability to detect individual differences (Carlson et al., 2014). Measurement tasks have ranged from multiple-choice questions on the GORT-4, to selecting a picture that matches a spoken word such as in the linguistic concepts sub-test of the Clinical Evaluation of Language Fundamentals-Fifth Edition (CELF-5) (Semel et al., 2013). Despite the widespread use of assessments such as these, they each measure different comprehension skills, which leaves scope for further research to identify a cohesive measurement system for assessing comprehension.

### ASD and Reading Difficulty

Research in ASD and reading difficulty supports the assertion of several profiles of reading ability in individuals with ASD (Davidson & Ellis Weismer, 2014; McIntyre et al., 2017, Grimm et al., 2017; Solari et al., 2019); typical reader, discrepant poor comprehender (above-average WR, significantly lower comprehension), below-average poor comprehender (average WR, below average comprehension), mixed-deficit (WR and comprehension below average), severe mixed deficit WR and comprehension well below average).

Existing data suggests that between 35 and 80% of samples of school-aged children with ASD display difficulties in one or more components of reading development, but that the most pertinent and impactful of these difficulties for academic development lies with underachievement in comprehension (Solari et al., 2017). While a 2004 meta-analysis of studies (McIntyre et al., 2017) found that targeted fluency instruction improved fluency and comprehension in typically developing students and those with reading disabilities, less was known about the relationship between fluency and reading comprehension for learners with ASD, since the majority of studies focus on variability in word decoding for these learners (McIntyre et al., 2017). In an effort to identify the impact that subcomponent reading skills have on reading comprehension for learners with ASD, Solari et al. (2017) conducted an investigation using structural equation modelling with the SVR as a framework. Their findings indicated that when fluency was added to the predictive model for reading comprehension it was the most pertinent predictor of comprehension performance for learners with high functioning ASD (HFASD), negating the effect of single word decoding and accounting for more variance than any other reading subskill in predicting comprehension performance.

Davidson (2021), conducted a review synthesizing empirical findings which use the DIER model as a framework with a broader sample of individuals with ASD, including those with language difficulties, and highlights that a deeper consideration across all aspects of WR, including text reading fluency will best inform literacy intervention for these learners. Video modelling may be a consistent method for targeting fluency deficits in children with ASD which could subsequently improve reading comprehension performance.

### Video Modelling

Video technologies may provide a useful tool for ensuring that modelling interventions requiring repetition are delivered consistently. VM integrates modelling and visually cued instruction to create an effective strategy for teaching new behaviours, which are generalised and maintained over time (Dowrick, 1999). Advancements in technology have increased the accessibility of these videos such that they can now be watched on a range of portable devices such as iPads or mobile phones, as well as through television or computer screens. Such technologies can be used to motivate engagement in productive behaviour and reduce problem behaviours in the classroom (Mechling, 2005).

VM has been used to teach social communication (Charlop & Milstein, 1989), play skills (Macdonald et al., 2009), self-help (Kuczera et al., 2016) and literacy skills (Kinney et al., 2003) to individuals with ASD. VM reduces the need for one-to-one social interaction that can be difficult for some individuals with ASD, and instead focuses on visual learning strengths (Stahmer et al., 2003). Additionally, studies have shown that VM may lead to faster acquisition of skills than in-vivo modelling for individuals with ASD (Allen et al., 2010; Charlop-Christy et al., 2000).

Research on reading fluency and VM intervention has utilised VSM procedures; a comprehensive search of the literature found just one peer-reviewed article that implemented a VM strategy with an alternative model to the self. Decker and Buggey (2014) compared VSM to VM with a peer model, examining the effect on fluency of elementary school students with learning disabilities. They found both strategies improved fluency, measured as WCPM. Although replication of effects was found across a multiple-baseline design, the lack of comparable research available limits the reliability of this study.

VSM has been heterogeneously applied to improve the skills of individuals with behavioural disorders (Lonnecker et al., 1994), developmental disabilities (Hitchcock et al., 2004), learning disabilities (Clare et al., 2000) and ASD (Kurnaz & Yanardag, 2018). In FFVSM an edited video is created that depicts the learner demonstrating a future skill that they are currently unable to demonstrate without additional support (Axelrod et al., 2014). Within a reading fluency intervention, a student may be filmed echoing single words or short phrases, and these clips are then edited together to achieve the illusion of fluent sentence reading. Although the evidence base is limited, studies have consistently demonstrated that FFVSM and VSM interventions can increase reading fluency for students with low reading abilities and learning difficulties (Decker & Buggey, 2014; Dowrick et al., 2006; Greenberg et al., 2002).

Robson et al. (2015) investigated the use of FFVSM for improving fluency and comprehension in 11 elementary students with low reading ability. All participants demonstrated improvements in accuracy, fluency and comprehension. FFVSM can be difficult to initially set up in an educational setting. This is due to the time and skills required for editing individual video models. As the individualised nature of a FFVSM intervention could be unsuitable for mainstream educational settings, teachers may find it easier to implement a VM intervention that can be used across multiple students, however, it could be a useful tool for students receiving one-to-one teaching or intervention in SEN settings. This research aims to explore the effectiveness of VM interventions for improving reading fluency in students with ASD, comparing FFVSM with VM using a teacher model. In addition, this research will investigate whether this method of improving reading fluency would impact student performance on comprehension questions. A secondary aim will consider the use of Talking Mats® as a method for obtaining student opinions on both forms of VM.

## Methods

A single-subject, alternating treatments design (ATD) was utilised for this research. The ATD (Barlow & Hayes, 1979), involved rapid alternation of the two interventions to analyse their effect on reading fluency. Reisener et al. (2014) recommended that future reading fluency research should examine the use of an ATD, as withdrawal designs may “overestimate results of both return to baseline and subsequent intervention phase conditions”. Similar to the withdrawal design, an ATD aims to control threats to internal validity by including a baseline phase; with an unstable baseline highlighting possible problems, such as maturation effects (Engel & Schutt, 2009). Multiple-treatment interference from sequential confounding and carryover effects can also threaten internal validity in ATDs. Wolery et al. (2010) suggested that intervention order must be randomly allocated to reduce multiple-treatment interference. Therefore, intervention order was randomly generated via computer software for this study. Additionally, intercomponent intervals were kept as long as possible to promote differential responding under the alternating conditions (McGonigle et al., 1987). As recommended by Holcombe and Wolery (1994), to increase external validity, the most successful intervention (when applicable) was solely continued following the alternating treatments phase to further evaluate its effectiveness.

Although multiple-baseline designs are also commonly used within VM literature (Powell & Gadke, 2018; Wu et al., 2018), an ATD appeared to be more ethical for this research. As no previous literature had compared the efficacy of FFVSM with VM using a teacher model, if one intervention proved to be more successful, an ATD would ensure all participants experienced this intervention.

To evaluate participants’ opinions on the interventions, an adaptation of Talking Mats® was used. ASD literature has often been criticised due to the absent views of the individual, with research heavily relying on parental views (Baric et al., 2015; Milton, 2012). This could be due to ambiguity surrounding methods for successfully eliciting opinions from children with ASD. Talking Mats as a facilitated conversation tool may reduce factors that can limit reliability and validity in qualitative research, such as questioning style or inadvertent prompting (Lewis, 2002). Talking Mats have previously been used to support individuals with disabilities when communicating about social activities (Germain, 2004), life plans (Cameron & Murphy, 2002), and challenging behaviour (Bradshaw et al., 2018).

### Participants

This study included four participants (three males, one female) between the ages of 8 and 15. All participants had a diagnosis of ASD and an Education Health and Care Plan (EHCP). In the UK, an EHCP follows an Education, Health and Care Assessment, and details the precise level and nature of support needed for children and young people under the age of 25 across education, health and social domains. Each participant had an approximated reading age lower than their chronological age, based on teacher reports and confirmed during initial assessments (See Table 1). All students attended the same specialist school in the south east of England and received individualised teaching based on the principles of ABA (see Table 1 for further participant characteristics; names are pseudonyms).

**Table 1:** Participant characteristics and teacher reported reading ability

Participant	Sex	Age (years)	Diagnosis	Approximate reading age (years)*	Corresponding ‘reading A–Z’ level	Teacher comments
Adam	Male	9	ASD and Epilepsy	5 to 6	aa to I	Verbal refusal to engage in reading tasks can be a precursor to problem behaviour
Liam	Male	8	ASD	6 to 7	E to P	Will often verbally refuse to engage in reading but continues without problem behaviour
Ciara	Female	14	ASD	6 to 7	E to P	Can become anxious and engage in behaviours such as crying if she finds a task too difficult
Daniel	Male	15	ASD	5	aa to I	Will often engage in off-task behaviour such as finger drumming but will continue reading task when instructed to do so

*Approximate reading age derived from teacher reports regarding which Stage ‘Oxford Reading Tree’ books students were currently reading at school

### Setting & Materials

Sessions were consistently conducted in the school gym. All participants were familiar with the location, and the detached nature of the building protected participant anonymity and offered a quieter environment in which to run sessions. All reading materials for this research were downloaded or adapted from Reading A–Z, a subscription service that provides online and printable texts. Reading A–Z texts are levelled, providing a wide range of stories, pitched at a wide range of reading ability (Klein, 2008). A sample of levelled texts (between 65 and 120 words in length) were randomly selected from a range of Reading A–Z resources, including benchmark passages, fluency passages and levelled books. To standardise these materials for this research, texts were converted into single-page documents with all pictures removed.

To assess reading fluency, running record sheets were used to record words read correctly, self-corrections made, and errors made by participants. Valanne et al. (2017) suggested that running records from Reading A–Z could provide a “detailed analysis of literacy growth when used systematically and accurately”. To assess whether any increase in fluency may have positively impacted comprehension, ‘quick check’ comprehension questions from Reading A–Z were used before and after the interventions. Five comprehension questions were presented which corresponded with benchmark passages. Three multiple choice answers were provided per question.

**Fig. 1:**
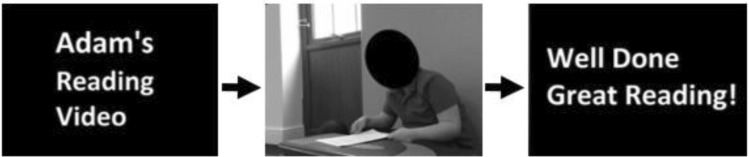
Example Screenshot from Participant VSM

A password encoded iPad was used to film sessions and to record footage for the video models. Recordings were transferred daily to a computer and stored within an encrypted location. To generate the video models, free editing software (Davinci Resolve) was used to cut, splice and alter the speed of frames until participants appeared to be reading at a fluent rate. Video models began with a black title page with white writing. A voiceover was included, stating: “reading video” (for the teacher model), or “[*name*]’s reading video” for the FFVSM (Fig. 1). All videos ended with a black page with white writing, a voiceover expressed: “well done, great reading!”.

Talking Mats visuals were created using the recommended talking mats framework suggested by Cameron and Matthews (2017) Visuals included a picture on a white background, with a caption in black writing below, e.g. “watching my reading video”. These visuals were presented as printed 7 cm squares.

### Target Behaviour

The target behaviour was reading fluency, operationalised as the ability to read aloud accurately and at a normative rate. Reading rate was measured by calculating words read correctly per minute (WCPM). The number of correctly read words was divided by the time taken to read the text (in seconds) and multiplied by 60. Accuracy was calculated as the percentage of words read correctly; number of correctly read words divided by the total number of words read, multiplied by 100.

### Independent (IV) and Dependent Variables (DV)

The IVs were two VM interventions, one using the teacher as a model, the other a FFVSM intervention where the participant acts as the model. Each participant experienced both independent variables with an ATD. The DV was WCPM on Reading A–Z levelled texts. As participants read a different text for each intervention session, reading materials were taken from their assigned levelled reading programme to ensure they were comparable.

### Measures and Data Collection

Running records were the primary measurement system used for this research. A typed running record was prepared to accompany each text, ready for the researcher to annotate as they viewed session videos. Each word was either coded as correct (tick), as an error (cross) or as self-correction (“SC”). Topographies of reading behaviour classified as errors included omitting a word, mispronouncing a word, substituting a word, and asking for help. If a participant requested assistance, the researcher immediately modelled reading the word correctly. In addition to fluency rate and accuracy, an error rate percentage was recorded for each running record. The number of errors made were divided by the number of words read and this was converted to a percentage (× 100).

Self-correction was recorded if a participant immediately re-read a word correctly following an error, or if they re-read a whole sentence correctly (including a word that had previously been recorded as an error). If a participant made a vocalisation whilst hesitating during reading (e.g. “um”), the next word was classified as self-correction. Self-stimulatory behaviours that did not present as a vocalisation (e.g. humming through the nose) were disregarded during coding. A self-correction rate percentage was calculated by dividing the number of self-corrected words by the total number of words read and this was converted to a percentage (× 100).

‘Quick check’ comprehension questions were used during the initial baseline, and at the end of the intervention. Data was recorded on the written question sheets by circling the participants correct answer. A comprehension score was calculated by dividing the number of questions answered correctly by the total number of questions and converting this to a percentage (× 100).

### Procedure

An initial reading assessment (using the Reading A–Z benchmark assessment) was conducted with each participant to identify an appropriate text level to be used for the experiment. A starting level was identified through discussions with teachers regarding the students approximate reading abilities. Each participant was asked to read a ‘benchmark passage’ whilst a running record was completed. The participant was then read five corresponding ‘quick check’ comprehension questions, with multiple choice answers to select from. If participants were able to read over 85% of the text accurately, at a rate below the average fluency expected for their age (Hasbrouck & Tindal, 2017), they remained at this text level. For participants reading at or above average fluency for their age, the text level was increased (towards an age-appropriate text level), until they were unable to answer over 20% of comprehension questions. From these assessments, it was confirmed that during the experiment, Adam and Daniel would read level F texts, Liam level G, and Ciara level I. These levels corresponded with the participants teacher reported approximate reading ages (see Table 1).

### Baseline

Three baseline sessions were conducted with each participant to establish stable measurement (Kazdin, 2010). The researcher explained what would happen during the session to each participant, e.g., “first you will read this page, then I will ask you five questions about what you have read” and waited until the participant confirmed (verbally) that they were ready to begin. The researcher placed the text (printed on one piece of A4 paper) on the table in front of the participant, signalling they could begin reading. In accordance with previous VM research (O’Kellems & Edwards, 2016), if the participant stopped engaging with the task for 5 s (e.g. had not read a word aloud) a prompt was provided. The researcher read the next word in the text as a verbal prompt for the participant to continue and counted this as an error.

When the participant finished reading, the researcher provided non-contingent social praise. Quick comprehension questions were administered following the first baseline session. Non-contingent social praise was also provided for answering these questions.

Rewards for each participant remained consistent across sessions; Adam received immediate access to an iPad for 5 min. Liam, Ciara and Daniel each received 2 tokens on their token-boards. This consistency would increase the likelihood that changes in reading fluency could be attributed to the interventions as opposed to differential reinforcement. Previous research has exemplified the impact of differential reinforcement within a choice-based antecedent intervention for increasing reading fluency (Daly et al., 2006).

### Alternating Treatments

The alternating treatments phase lasted for 10 sessions, with approximately 24-h intervals between sessions. Due to unforeseen absence from school, Adam experienced a 5-day interval between the first and second intervention sessions. He also experienced a convulsive seizure between sessions 8 and 9 which was not typical for him, this required the administration of Buccal Midazolam medication. During each session, the participant experienced one of the two interventions. Intervention order was randomly generated using a freely available web program.

### Video Modelling Intervention (Teacher Model)

Procedures in this condition were similar to those in the second and third baseline sessions, with the addition of the following task: before being asked to read a text, participants were instructed to watch the teacher video model. A classroom teacher, familiar to all participants, was used as the model. The video was presented on an iPad, placed in front of the participant. Three versions of this video were created, with the teacher reading the students’ assigned levelled text. The teacher read for all students at an average rate of 202 WCPM with 100% accuracy.

### Feedforward Video Self-Modelling Intervention

Procedures in this condition were identical to the VM intervention, with the participant acting as the model in each video. Participants were shown an edited video of themselves reading with 100% accuracy. Adam’s video model showed him reading at 161 WCPM (83 WCPM above his baseline assessment for the same text). Daniel’s video model showed him reading at 116 WCPM (47 WCPM above baseline), Liam’s video model showed him reading at 129 WCPM (69 WCPM above baseline). Ciara’s video model showed her reading at 163 WCPM (35 WCPM above baseline). All video model durations were between 43 and 55 s.

## Best-Treatment or Follow-Up

Following the alternating treatments, each participants’ mean WCPM was calculated for each intervention and compared to their mean baseline WCPM. To calculate a mean score, WCPM were totalled and divided by the number of sessions. This comparison was used to identify the most successful intervention, which was then solely continued for three further sessions in a ‘best-treatment’ phase to increase external validity of the study Holcombe and Wolery (1994). If neither intervention demonstrated an improvement in WCPM for any participants, one follow-up session was conducted following baseline one procedures.

### Maintenance and Generalisation

For participants who had improved their reading fluency during the intervention, four further sessions were conducted to assess for maintenance and generalisation. A recent review of ABA literature found no clear guidelines to determine how much time should pass before behaviour is considered ‘maintained’, with researchers waiting one day to 5 months (Pennington et al., 2018). For this study, 26 days elapsed between the best-treatment and maintenance phases. To measure whether increased reading fluency had maintained, participants repeated the readings from the best-treatment phase. To test for generalisation across time and stimuli, the same procedure was carried out for a novel levelled text from Reading A–Z.

### Talking Mats (Qualitative Interviews)

During a final session with the researcher, each participant was invited to express their opinions on various elements of the intervention during a Talking Mats interview. The participant sat adjacent to the researcher on a mat and the researcher explained that they were going to talk about the reading experiment and laid out three heading visuals: “I like it”, “I don’t like it” and “I don’t know”. All participants had previous experience of using Talking Mats for different topics. Different elements of the intervention (e.g. setting, materials, different types of models, and reinforcement) were displayed on 12 visual cards. The researcher gave one picture at a time to the participant and read aloud the caption (e.g. “watching Adam’s reading video”). The participant was given 30 s to respond by placing the visual under one of the headings. If no response was made, the researcher reminded the participant to choose where they wanted to put the picture, then repeated the caption. If no response was made after a further 30 s, the visual was removed and the next visual was presented. When the participant placed a visual under a heading, the research probed for a reason for the opinion, e.g. asking: “why do you like reading?”. If the participant did not respond within 10 s, the researcher presented the next visual. When all visual cards had been discussed, a photograph was taken of the Talking Mats to supplement video documentation of the conversations. Video recordings were used to monitor threats to reliability and validity, including interviewer influence and contextual issues.

### Data Analysis

In addition to descriptive statistics, data were graphically displayed to enable visual analysis of trends and variability. To support this analysis, an effect size was measured by calculating percentage of nonoverlapping data points (PND) for WCPM during the alternating treatment and best-treatment phases. Jenson et al. (2007) reported that PND is commonly used within single-subject research to demonstrate the effectiveness of interventions. PND scores above 90 demonstrate very effective interventions, 70 to 90 validate an effective intervention, scores of 50 to 70 reflect uncertainty, and interventions scoring below 50 are ineffective (Scruggs & Mastropieri, 2001).

### Reliability

To evaluate the measurement system and ensure the target behaviour was clearly defined, trial-by-trial inter-observer agreement was calculated for words correct, errors, and self-corrections. Inter-observer agreement was calculated for 20% of intervention sessions (two for each participant). A Board Certified Behaviour Analyst (BCBA) viewed the intervention session videos and coded words on running record sheets (according to the operational definitions provided by the researcher). Trial-by-trial inter-observer agreement between the researcher and a second observer was calculated at 97%. To evaluate researcher objectivity, intra-observer agreement was calculated between initial video coding on running records, and repeated coding ten weeks later. The researcher re-watched videos of the intervention sessions and completed running records to compare with the originals. The researcher scored 20% of intervention sessions and calculated an intra-observer percentage. Intra-observer agreement was calculated at 99%.

## Results

### Reading Fluency

Mean WCPM for baseline, intervention and post-intervention phases are displayed for all participants in Table 2. For two participants (Ciara and Adam), a reduction in reading fluency across both interventions during the alternating treatments phase resulted in a discontinuation of both interventions and subsequently no best treatment phase for these participants.

**Table 2:** Words correct per minute

Participant	Data type	Baseline	During VM (ATD)	During FFVSM (ATD)	During FFVSM (best treatment)	Post-intervention (follow-up or generalisation probe)
Liam	Mean	52.3	47.8	58.4	62.3	61
	Median	55	41	60	60	61
	Range	42–60	37–72	47–67	58–66	61
Daniel	Mean	65.3	74.2	74.4	79.3	62
	Median	66	78	70	71	62
	Range	61–69	67–80	65–95	65–102	62
Ciara	Mean	134	124.3	128.5		157
	Median	135	121.5	136.5		157
	Range	128–139	95–159	99–142		157
Adam	Mean	96	81.8	75.2		62
	Median	102	87	83		62
	Range	78–108	47–116	56–91		62

### Liam

**Fig. 2:**
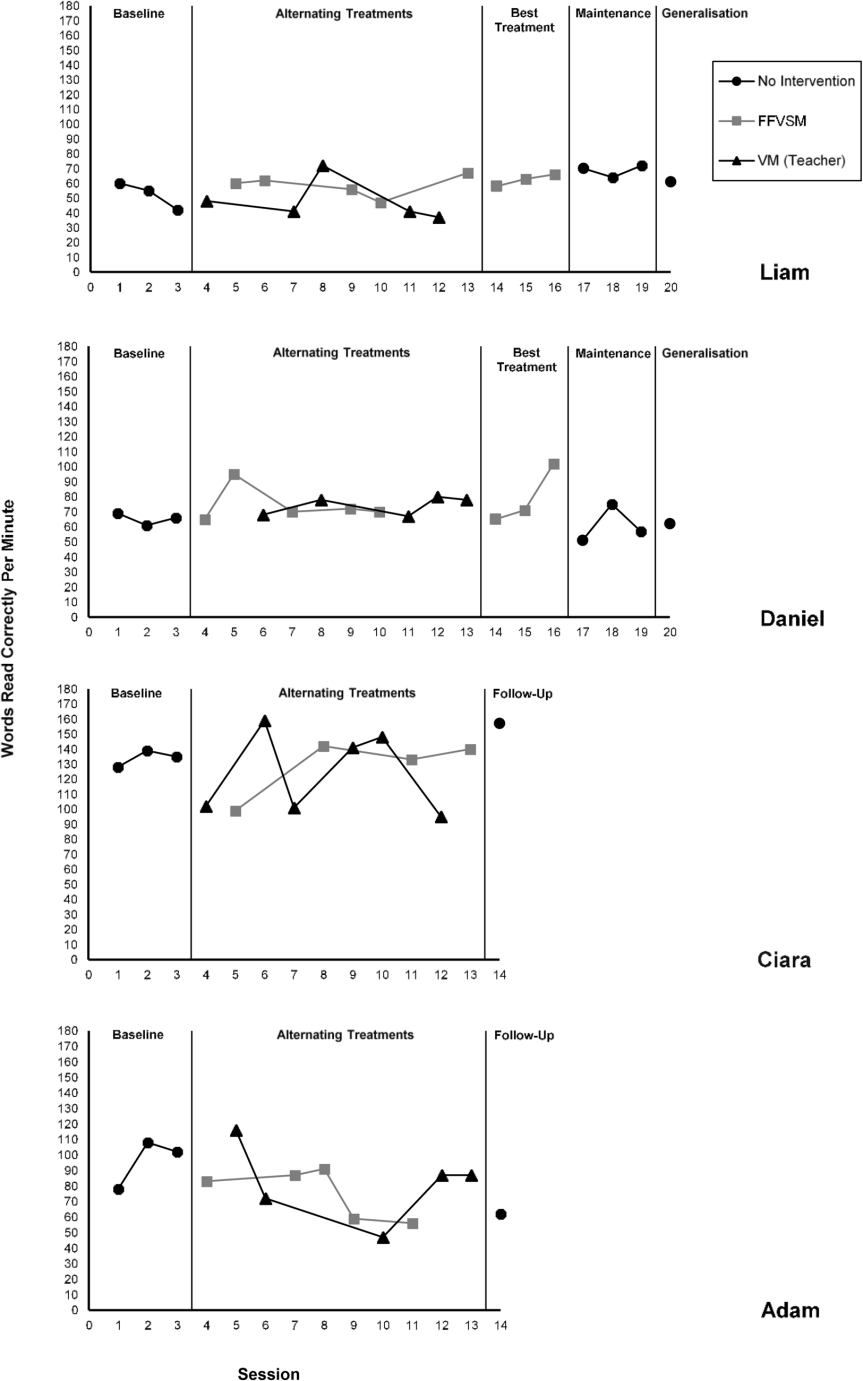
Reading Fluency Scores (WCPM) Across Each Condition: Baseline, Alternating Treatments (FFVSM Versus VM With Teacher Model), Best Treatment (FFVSM), Maintenance, and Generalisation

As illustrated in Fig. 2, during baseline Liam’s mean WCPM score was 52.3, 59.7 WCPM below the expected average for his age (Hasbrouck & Tindal, 2017).

During the alternating treatments phase, the VM intervention using a teacher as a model was found to be ineffective for Liam. There was a slight continuation of the declining trend seen during baseline, with reading fluency reducing from a mean of 52.3 WCPM (range: 42–60) to 47.8 WCPM (range: 37–72) during the intervention. Although the VM intervention correlated with the highest increase in reading fluency during one session (72 WCPM), the PND from baseline to VM was only 20%, indicating that overall, the intervention was ineffective.

In comparison, at the final FFVSM session; Liam’s reading fluency increased by 8 WCPM when compared to the final baseline session. Visual comparison between the two treatments indicates that FFVSM was more effective for Liam; his reading fluency increased to 58.4 WCPM (range: 47–67) during the alternating treatments phase. However, during this phase of the experiment, the effectiveness of the intervention was questionable as the PND for FFVSM was only 40% (with only two of five data points exceeding the highest baseline score). The effect size improved to 67% when FFVSM was the sole intervention continued during the best treatment phase, and Liam’s reading fluency further increased to a mean of 62.3 WCPM (range: 58–66). When the three texts from the best treatment phase were re-assessed five weeks later to evaluate whether the intervention effects were maintained, Liam was able to read these passages more fluently (mean fluency: 68.7 WCPM, range: 64–72). Although his reading fluency was slightly lower when reading a novel text (61 WCPM), this still demonstrated a generalised improvement in fluency compared to baseline (mean 52.3 WCPM).

### Daniel

Daniel made the greatest improvement in his reading fluency during the FFVSM intervention (Fig. 2), averaging 74.4 WCPM (range: 65–95), compared to a mean of 65.3 WCPM during baseline. However, the FFVSM intervention only produced an increase of 0.2 WCPM compared to the VM intervention. The PND for FFVSM during the alternating treatments phase compared to baseline was 80%, demonstrating that this intervention was more effective for Daniel than it was for Liam. In contrast to Liam’s results, during the best treatment phase, the PND for Daniel’s reading fluency scores reduced (to 67%). However, visual analysis of the data revealed a strong upward trend in performance during this condition (from 65 to 103 WCPM). The PND for the VM intervention was 60%. During maintenance sessions Daniel’s scores were unstable, with his mean WCPM reducing to 61, 4.3 WCPM below his mean baseline score. Similarly, he did not demonstrate generalisation of improved reading fluency to a novel text; this was read at a rate of 62 WCPM.

### Ciara

Overall, FFVSM was found to be ineffective for Ciara, with mean fluency reducing to 128.5 WCPM and PND calculated at 50%. Similarly, reading fluency decreased to a mean of 124.3 WCPM during the VM intervention, with PND also at 50%. Performance during this intervention was highly variable, with scores ranging from 95 to 159 WCPM. As neither intervention produced stable improvement in reading fluency for Ciara, no best treatment phase was conducted. One follow-up session assessed performance after the interventions had ended. Ciara read a novel text with 98% accuracy at an increased fluency level of 157 WCPM (6 WCPM above the average norm for her age).

### Adam

Adam had an ascending baseline, which is reflective of improvement that would be expected due to regular teaching instruction. During baseline, he correctly read between 78 and 108 words per minute, with a mean of 96. When the FFVSM intervention was introduced there was an immediate decline in reading fluency, and this trend continued throughout the intervention except for one session. With PND at 0%, it is clear this intervention was ineffective for Adam; and mean reading fluency during the intervention was 75.2 WCPM (range: 56–91), a reduction of 20.8 from the baseline mean. The video modelling intervention using a teacher model was similarly ineffective (PND: 20%), with Adam demonstrating a mean reading fluency of 81.8 WCPM (range: 47–116). Adam initially improved his reading fluency during the first VM session, however there was a rapid decline during the following two VM sessions. Performance increased and stabilised towards the end of the intervention, but the rate remained below the baseline mean.

During the follow-up session, when both interventions had finished, reading fluency further declined to 62 WCPM. The decline in performance across this research may have been influenced by unexpected confounding variables. During the first two sessions of the intervention Adam was absent from school due to illness. Between sessions 8 and 9, Adam experienced a prolonged convulsive seizure at school requiring administration of Buccal Midazolam, which has sedative properties. This was the first time Adam had experienced a convulsive seizure at school, and historically these occurred infrequently at home (once or twice a year).

### Reading Comprehension

Two out of four participants (Daniel and Liam) showed increases in their comprehension scores, with scores increasing by 40% (2 additional correct responses) and 20% (1 additional correct response) respectively. While Adam’s fluency scores decreased across both interventions, his comprehension score remained at 20%. Although Ciara’s fluency scores improved during follow-up, they were highly variable throughout both interventions and she was unable to correctly answer any of the post-intervention comprehension questions (having previously scored 20% prior to the interventions).

### Social Validity

**Fig. 3:**
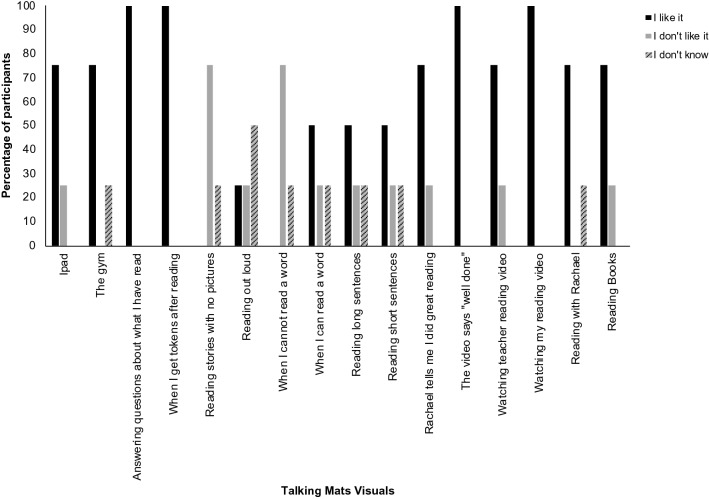
Graphical Display of Participant Responses During Talking Mats Interviews

All participants reported that they enjoyed watching their own reading video (during the FFVSM intervention). The majority of participants (75%) also stated that they liked watching the teacher reading video used during the VM intervention. Adam had sorted this picture into the “I don’t like it” pile. All participants said that they liked the social praise, which was provided at the end of the video models during both interventions. Most participants also said that they liked: ‘when Rachael tells me I did great reading’, with Ciara explaining: “It’s encouraging”. Liam, Daniel and Ciara who had earned tokens as non-contingent positive reinforcement for reading during this study, all expressed that they liked receiving these (Fig. 3).

Making errors when reading was viewed negatively, with 75% of participants expressing that they did not like it when they were unable to read a word; Ciara exclaimed: “I don’t like being stuck with words” as she sorted this visual. Most participants also said that they did not like ‘reading stories with no pictures’. During this research, texts had been formatted to be as similar as possible, which meant no images were used. Participants had conflicting opinions regarding other elements of the texts, such as sentence length. Liam said that he did not like reading long sentences because “it takes too long”. In contrast, Ciara said she does not like short sentences, and elaborated: “I prefer reading long sentences”. As Liam and Ciara were more experienced in using Talking Mats to discuss a range of topics, to extend their interviews, they were asked to sort the texts they had read into: “it was easy”, “it was hard”, or “I’m not sure”. Ciara, who had responded predominantly positively about reading during the initial interview, sorted all passages into “it was easy”. In comparison, Liam categorized 9 of the passages as hard and said he was not sure about the rest.

## Discussion

Previous research on FFVSM and reading fluency has focused on improving the skills of individuals with learning disabilities, (Decker & Buggey, 2014; Dowrick et al., 2006), or low reading ability (Robson et al., 2015). The improvements in fluency experienced by Liam and Daniel in this study, demonstrate that findings of previous FFVSM research can be replicated for students with ASD. In a comparison of PND scores the FFVSM intervention was found to be statistically more effective for Daniel (80%), than for Liam (40%). Although Liam’s PND score suggests the intervention was ineffective, an immediate increase in his fluency was seen when this intervention was implemented, and when the intervention was solely continued during the best treatment phase PND increased to 67 (as did Daniel’s score), similar to findings by Edwards and Lambros (2018) who found a moderate intervention effect (60%) for FFVSM.

PND was selected to demonstrate the effectiveness of interventions within this study, as conventional effect size could have exaggerated results within the best treatment phase due to the minimal number of data points (Scruggs & Mastropieri, 2001). Bellini and Akullian (2007) used PND in a meta-analysis to examine the effectiveness of video modelling interventions for individuals with ASD and concluded that all VM procedures (including FFVSM) are effective for promoting acquisition of skills. The results of this study unfortunately do not support these findings. While Daniel’s fluency scores increased with both VM and FFVSM interventions, Liam only experienced fluency improvement with FFSVM while Adam and Ciara did not experience improvements in fluency with either intervention.

Bellini and Akullian (2007) also reported that results from VM and VSM “are maintained over time and transferred across persons and settings”. Liam’s results supported these findings; he maintained the improved level of fluency once the intervention had concluded and demonstrated generalisation when reading a novel text. In comparison, Daniel’s fluency during maintenance testing reduced to below baseline levels and no generalisation occurred. This disparity in maintenance effects may have resulted from differences in the way reading behaviour was reinforced post-intervention. During the FFVSM intervention, both participants were provided with two tokens when they had finished reading a text (non-contingent on how fluently they had read). Following the intervention, participant’s teachers had not been instructed to continue this level of reinforcement. As students utilised token economy systems, with tokens provided on a variable ratio schedule, reinforcement for fluent reading may have been unintentionally thinned too quickly (Volkert et al., 2009).

Although FFVSM was found to be an effective intervention for two participants in this study, conflicting results were found for Adam and Ciara. In Adam’s case, this decrease may have been as a result of a prolonged convulsive seizure at school that required administration of midazolam which has been found to impact on learning, memory and sustained attention (Hsu et al., 2015; Jackson et al., 1993); potentially explaining Adam’s larger decrease in fluency seen between sessions 8 and 9. While Ciara’s fluency increased, high levels of variability in her fluency scores meant a functional relationship between either intervention and the dependent variable could not be identified. Therefore, it must be said that contrary to previous findings (Bray et al., 1998; Decker & Buggey, 2014), FFVSM may not be an effective intervention for every student, in particular those with ASD.

### VM and Reading Fluency

Based on previous research (Ardoin et al., 2009; Kinney et al., 2003; McCoy & Hermansen, 2007; Stevens et al., 2017; Welsch, 2007), it was hypothesised that a VM intervention using a teacher model would increase fluency for all students in this study. Results contradicted this hypothesis, as three participants mean fluency scores reduced during the VM intervention compared to baseline. Creating an adult video model may be more time efficient than a peer or self-model (Ayres & Langone, 2007), and most studies report that the type of model does not influence the effectiveness of VM interventions for teaching ASD students new skills (Ihrig & Wolchik, 1988; Nikopoulos & Keenan, 2003; Wang & Koyama 2014). However, the findings of this study support the argument that student learning is most efficient when they observe a model that resembles themselves (Buggey et al., 1999; Thoresen & Hosford, 1973) as more participants made improvements with their fluency during the FFVSM intervention. Furthermore, session notes demonstrated that one participant (Liam) quickly developed a preference for his FFVSM video, and when presented with the teacher video would request: “can I watch my reading video instead?”. This does not support previous reports that students with ASD display no preferences regarding which type of video model they watch (Mechling & Moser, 2010). However, Liam’s theorised preference is subjectively based on his comments; further research would be required to explore this objectively perhaps through the use of a concurrent-chains schedule (Hanley, 2010) to determine how frequently participants would select FFVSM over VM.

### Relationship Between Fluency and Comprehension

The extant literature suggests that fluency skills are a strong predictor of comprehension skills (Kim, 2020; Solari et al, 2017) and that targeted fluency instruction improved fluency and comprehension skills in typically developing learners, however, there was not enough conclusive evidence in this study to support that targeted fluency instruction will positively impact comprehension for learners with ASD. Ciara’s fluency score during the follow-up session had increased compared to baseline, but her comprehension had decreased, and she was unable to answer any questions correctly. Adam’s fluency declined throughout the study, but his comprehension score remained consistent. While Liam and Daniel both demonstrated increased fluency and improved their comprehension scores during this research, further experimentation would be required to examine whether there was a causal relationship between these variables.

Davidson’s review (2021) indicated that a deeper consideration of performance across all aspects of WR would best inform literacy intervention for learners with ASD. The current study only considered the impact of text reading fluency, without any targeted instruction on the remaining components of WR (single word decoding, single nonword reading and text reading accuracy) as an intervention. This has potentially resulted in the observed variability in findings within this study.

The inconsistent relationship between fluency and comprehension within this study could be due to the measurement system used. Fluency was measured using WCPM which only accounts for rate and accuracy of reading despite some research stressing the importance of the relationship between prosody and comprehension (Kuhn & Stahl, 2000). Research has demonstrated that prosody may contribute more significantly to comprehension than rate or accuracy, and that comprehension is most accurately predicted when all three of these factors are combined (Valencia et al., 2010). The Multi-Dimensional Fluency Scale (Zutell & Rasinski, 1991) may have provided a more accurate measurement of fluency, however, this assessment is not well designed for the ASD population. An in-depth scope of the literature for the current study resulted in very few options of fluency assessments, which include prosody as a component and are applicable for use with learners with ASD. Similarly, replication of this research would benefit from the addition of a standardised reading assessment which provides comparative norms for comprehension, such as the GORT-5.

### Social Validity

Behaviour analysts aim to conduct applied research that will produce socially significant outcomes (Baer et al., 1968). Social validity should be measured to ensure goals, procedures, and intervention results are considered acceptable, in particular by the client (Wolf, 1978). A recent systematic literature review of single-subject case research found just 26.8% of 429 articles included an assessment of social validity (Snodgrass et al., 2018). This could be due to the lack of available guidance on how to measure social validity, or the increased time and costs associated with completing additional assessments. One common method of assessing social validity is to measure opinions about interventions using rating scales or questionnaires (Carroll & St. Peter, 2014); in ASD research these have been commonly administered to parents, somewhat neglecting opinions of the children receiving the intervention (Baric et al., 2015). This study assessed social validity by obtaining the views of children with ASD, through visually supported Talking Mats interviews.

Talking Mats interviews were useful for assessing whether either of the interventions was viewed more favourably; and for exploring the acceptability of intervention elements (e.g. opinions on the length and format of texts). All participants reported that they liked watching the FFVSM video supporting the claims of previous research (Edwards & Lambros, 2018). The majority of participants expressed that they liked receiving social praise and tokens for reading, highlighting the importance of including reinforcement procedures when implementing antecedent interventions (such as VM). The interviews also revealed which aspects of the intervention participants disliked; for example, reading stories without pictures, and when they were unable to read a word. Standardised printed texts were chosen for consistency within this study, and in accordance with previous research (O’Kellems & Edwards, 2016), prompts were only provided if participants had stopped reading for 5 s. Future research could eliminate the unpopular elements of the FFVSM intervention by incorporating errorless teaching practices and including illustrated texts which could increase motivation (Brookshire et al., 2002).

### Limitations

Whilst the current study offers important contributions to the literature on reading fluency and VM, consideration must be given to several limitations when interpreting these findings. Firstly, this research employed a single subject design, which can lack generalisability and external validity (Alnahdi, 2015). It has been suggested that replication of intervention effects across multiple participants within single subject research demonstrates generalisability (Simonsen & Little, 2011), however positive intervention effects were only replicated for half of the participants in the present study. Ciara made overall improvements with her fluency but due to high variability during the interventions no functional relationship could be determined. This could have been the result of multiple treatment interference, a common problem with ATDs (McGonigle et al., 1987).

Secondly, although Reading A–Z reading passages have been used in previous fluency research (Bridges, 2008), and were selected to accommodate participants with a wide range of reading abilities, researchers have argued that levelled passages are not entirely equal (Francis et al., 2008). As identical Level F passages were read by both Adam and Daniel during this research, their data was further examined and highlighted possible extraneous variables that may have influenced results. Both participants displayed similar patterns of performance across the texts, for example an identical sharp increase in fluency is seen between sessions 4 and 5, with Adam’s score increasing by 33 WCPM and Daniel’s score increasing by 30 WCPM. This similarity occurred despite the participants experiencing different interventions during these sessions. A noticeable correlation was also evident for both Adam and Daniel between fluency and the number of words in the text (Level F texts ranged from 65 to 110 words). Both participants demonstrated a similar decline in fluency as the text length increased. This supports previous research that increased passage length (or duration of timed reading) may negatively impact fluency (Daane et al., 2005). Similarly, Barth et al. (2014), reported that 55% of variation in fluency was caused by variation in text-level features, such as passage length and genre.

A third limitation occurred due to the individualised nature of the FFVSM intervention, which meant it was not possible to control some confounding variables across participants, including the length of the video, and the speed of words read correctly per minute during the self-model. A literature review discussed that video length may affect the level of attending for individuals with ASD (Lee, 2015), but suggested more research is required to explain these findings. Video models were also only watched once per session during the present research; although this was adequate for producing improvements for some participants, research has shown that multiple viewings may be more beneficial for students with ASD (Wert & Neisworth, 2003; Wang & Koyama, 2014). Furthermore, beyond their diagnosis of ASD, very little neuropsychological information was known about the participants in this study. To ensure adherence with the ethical approval obtained from their host institution, the authors did not have consent to gather, or report on neuropsychological information beyond that which is reported in Table 1. This limits the extent to which the current data can be considered useful, given how the variability in profiles of individual children with ASD and how this variability is a confounding factor in influencing the effectiveness of VM as an intervention.

A further extraneous variable that may have had a confounding effect on fluency, was participants’ prior experience of reading interventions. Three participants had previously engaged with the Headsprout Early Reading Program, which incorporates strategies such as clearly defined mastery criterion, errorless teaching and guided practice (Storey et al., 2017). Previous research has demonstrated that students can improve their reading skills (including fluency) using the Headsprout Early Reading Program (Clarfied & Stoner, 2005; Storey et al., 2019), and it is therefore possible that this prior experience could have affected student performance in the present study. Daniel, who had not previously experienced any online reading interventions, was the only participant to demonstrate fluency improvements during the teacher VM intervention; he also exhibited the largest improvement during the FFVSM intervention.

It is important to highlight that continuous reading during intervention phases, and repeated reading during the maintenance phase could have accounted for the improvements in fluency during this study. During the current study, participants read a range of novel fiction and non-fiction texts and may therefore have improved their reading skills due to more frequently accessing a wider range of materials. During the maintenance phase, students repeated texts they had read during the best treatment phase, and any improvement could therefore have been influenced by this repetition (Stevens et al., 2017).

Finally, in respect to assessing reading comprehension, this research utilised multiple-choice comprehension questions; it is therefore possible that participants answers were not entirely passage dependent. This has been a major criticism of similar reading comprehension tools such as the GORT-4 (Keenan & Betjemann, 2006), which has since been updated (GORT-5) to ensure passage dependency through the use of open-ended questions.

### Practical Applications and Future Research

Contrary to previous research, improvements were not observed for all participants; additional research is therefore recommended to further explore the use of FFVSM to improve the reading skills of ASD students. In addition, creating individualised video models can be time consuming so it could be argued that FFVSM is best suited for use within individualised teaching (e.g., special education units, or ABA schools offering one-to-one support).

Within mainstream settings, a more feasible intervention for teachers to implement would be a video of themselves modelling fluent reading. However, results from this research suggest this may be ineffective for most students with ASD. As this result may have been influenced by multiple treatment interference, and there are no previous studies to act as a comparison, future research should utilise other methodologies (e.g., a multiple-baseline design) to replicate this research with a larger number of participants. Research could also explore the use of VM procedures for improving the fluency of a wider range of students (e.g., in mainstream schools, or with dual language learners).

Similarly, the use of Reading A–Z resources with ASD students had not been previously explored in the academic literature. Reading A–Z provide levelled fluency passages, correlating comprehension questions, and running records for measuring fluency. These resources were found to be beneficial for recording and reporting on fluency and were accessible for ASD students who were reading at an approximate age level of 5 to 7 years. Furthermore, teachers have reported that following this research, some participants have continued to use the printable levelled books from Reading A–Z to further expand their reading skills. Future research could examine how these resources can be incorporated into reading instruction or interventions for students with ASD.

Replication of this study should aim to address some of its limitations. For example, it would be useful to explore the impact of text-level features, such as passage length, or whether there is a difference between fluency when reading fiction versus non-fiction texts. Elements of the video modelling intervention could also be further manipulated, for example, examining the effectiveness of more frequent video viewings (Wang & Koyama, 2014). Additionally, research could investigate the application of FFVSM interventions for teaching other reading skills to students with ASD, such as decoding or recognising sight words. This would also expand upon the findings of Ayala and O’Connor (2013), who used VSM to increase these reading skills for students with low reading abilities.

A final recommendation for future research is to focus on exploring more ways to include individuals with ASD within the social validation process. This research has demonstrated that the Talking Mats framework can be a useful tool for enabling school students with ASD to provide their opinions on interventions they have experienced. Regardless of the tool that is used, improving the number of studies which assess social validity should be a priority area of development for behaviour analysis.
